# Antibiotics for fever in patients without perforation after gastric endoscopic submucosal dissection and endoscopic submucosal excavation may be unnecessary: a propensity score-matching analysis

**DOI:** 10.1186/s12876-021-01602-1

**Published:** 2021-02-12

**Authors:** Yongkang Lai, Qian Zhang, Xiaolin Pan, Zhenhua Zhu, Shunhua Long, Xiaojiang Zhou, Guohua Li, Yin Zhu, Youxiang Chen, Xu Shu

**Affiliations:** Department of Gastroenterology, The First Affiliated Hospital of Nanchang University, 17 Yongwaizheng, Street, Nanchang, 330006 Jiangxi Province China

**Keywords:** Endoscopic submucosal dissection, Gastric lesions, Antibiotics, Fever, Propensity score-matching analysis

## Abstract

**Background:**

Endoscopic submucosal dissection (ESD) and endoscopic submucosal excavation (ESE) have been widely used and have gradually become the main endoscopic treatment for gastrointestinal mucosal and submucosal lesions. Whether antibiotics are necessary for fever after gastric ESD and ESE remain unclear. The aim of this study was to analyse the value of using antibiotics in patients without perforation after ESD or ESE with fever.

**Methods:**

In this retrospective study, patients with fever without perforation after ESD or ESE from January 2014 to January 2019 were included and divided into 2 groups: the antibiotic group and the non-antibiotic group. Fever and hospitalization time were compared between the 2 groups after propensity score matching.

**Results:**

Overall**,** 253 patients meeting the inclusion criteria were enrolled in the present study, with 186 patients in the non-antibiotic group and 67 patients in the antibiotic group before matching, 55 patients in the non-antibiotic group and 55 patients in the antibiotic group after matching with all baseline characteristics balanced (*p* > 0.05). The duration of fever was not significantly different between the 2 groups (*p* = 0.12). However, the median hospitalization stay in the antibiotic group was longer than that in the non-antibiotic group (8 vs 7, *p* = 0.007).

**Conclusions:**

Antibiotics may be unnecessary for fever in patients without perforation and without serious co-morbidities after gastric ESD or ESE.

## Background

Endoscopic submucosal dissection (ESD) and its derived technique, endoscopic submucosal excavation (ESE), have been commonly used as minimally invasive treatment for gastric lesions [1–3]. Compared with conventional surgical treatment, ESD and ESE are associate with shorter operative times, shorter hospital stays and lower complication rates [4, 5], and long-term survival appears equivalent between ESD and gastrectomy patients [6]. However, ESD and ESE are associated with some complications, such as perforation, bleeding, and pyrexia. Among them, pyrexia is a common complication after ESD or ESE, and previous study have reported that fever occurs in 19.5% of cases [7]. Bacteraemia, exposure of large wounds and long operation times may be correlated with pyrexia in patients treated after ESD or ESE [7–9]. However, the exact mechanism still needs to be investigated further.

At present, it is almost a consensus that there is no indication for prophylactic antibiotics after gastric ESD or ESE. Many studies have shown that although continuous submucosal defects caused by ESD or ESE may increase the risk of bacteraemia and/or endotoxaemia, the rate of bacteraemia after procedure remains low or the bacteraemia is transient. Therefore, the researchers concluded that prophylactic antibiotics might not be necessary for patients after ESD or ESE [8–12]. However, these studies only focused on whether antibiotics were used in advance after ESD or ESE. It remains uncertain whether antibiotics are necessary for patients with fever after gastric ESD or ESE, especially for those without perforation during or after procedure, since post-ESD bacteraemia is transient and the use of antibiotics will increase the cost of hospitalization. Relevant research is rare. Hence, the purpose of this study was to analyse whether fever after ESD or ESE should be treated with antibiotics in patients without perforation.

## Methods

### Participants

This is a retrospective study performed at the Department of Gastroenterology, the First Affiliated Hospital of Nanchang University in China. Patients who underwent ESD or ESE for gastric lesions at our department between January 2014 and January 2019 were enrolled. Informed consent was obtained from every patient. The exclusion criteria were as follows: (1) did not have a body temperature exceeding 37.5 °C after ESD or ESE (regardless of the duration of the fever period); (2) age younger than 18 years or older than 85 years; (3) the use of antibiotics within 2 weeks before ESD or ESE; (4) immunodeficiency status; (5) serious cardiovascular, cerebrovascular, or hepatorenal diseases; (6) fever (temperature > 37.5 °C) before the procedure; (7) incomplete demographic data; and (8) patients with perforation during or after the procedure.

### Relevant definitions

Intraoperative bleeding refers to any bleeding in which haemoglobin is diluted from preoperative level to a level > 2G/dl the day after ESD or ESE. Perforation is defined as other organs, extraluminal fat, or extraluminal space outside the muscle layer that can be seen through endoscopy during the ESD or ESE procedure, regardless of air accumulation in the abdominal cavity, retroperitoneum or mediastinum [13]. En bloc resection is defined as the endoscopic removal of a lesion in one piece and the acquisition of a single specimen. Procedure time is defined as the period from intraoperative marking time to withdrawal time. Fever is defined as a temperature > 37.5 °C after the procedure (regardless of the duration of the fever period). Because the fever time could not be accurately recorded in minutes, we recorded fever duration < 1 day, > 1 day ≤ 2 days, > 2 days ≤ 3 days, > 3 days ≤ 4 days, and > 4 days ≤ 5 days as 1, 2, 3, 4, and 5 days, respectively.

### Gastric ESD and ESE procedure

Before the ESD procedure, patients underwent an endoscopic ultrasound (EUS) test with a radial-scanning echo endoscopy unit (UM240; Olympus Co., Ltd., Tokyo, Japan) or a 12-Fr catheter probe (UM-3R, 12 MHz; Olympus Co., Ltd., Tokyo, Japan) to identify the size, shape and layer of origin of the lesion. In addition, abdominal computerized tomography (CT) was performed to evaluate the tumour location, growth pattern (intra/extraluminal) and the possibility of lateral growth or distant metastasis. All ESD and ESE procedures were performed by experienced endoscopists with more than 10 years of experience. A single-channel endoscope (GIF-Q260J; Olympus Co. Ltd, Tokyo, Japan) was used in this procedure. After intravenous anaesthesia with propofol, routine vital sign monitoring was performed. After identifying the gastric lesion through endoscopy, dots were marked around them with argon plasma coagulation (APC, 40 W soft coagulation). Then, 250 ml glycerol fructose, 2–3 ml indigo carmine and 1 ml 1:10,000 epinephrine were injected into the submucosal layer to elevate the lesion. The superficial mucosa was incised along the outer edge of the marker point by endoscopists using a hook knife (KD 620LR, Olympus). Subsequently, an IT knife-2 (KD 611L, Olympus) was used to gradually separate the submucosal layer and lesion, and a snare (SD-230U-20; Olympus) was used to help with the removal of the lesion if necessary. If the gastric lesions originating from submucosal layer or superficial muscularis propria (MP) layer ESE was used. ESE is the derivative technology of ESD. On the basis of ESD technology, continue to gradually peel off the submucosa and part of the muscularis propria at the base of the tumor. Hot biopsy forceps (FD-410LR; Olympus) or argon plasma coagulation (APC 300, ERBE) were used for intraoperative haemostasis. If there was active perforation caused by tumour excavation, titanium clips (HX-610-135; Aomori Olympus) or an over-the-scope clip system (OTSCs, Ovesco Endoscopy AG, Tübingen, Germany) were used to close the perforation. After removing the lesions, a stomach tube was placed based on the experience of endoscopists to reduce gastric pressure for at least 24 h. All specimens were measured and immersed in formalin and were sent to the pathology department for immediate identification of the nature of the lesion.

### Postoperative management

Patients were sent to our ward after recovery from anaesthesia and were asked to fast for 2–5 days. All of the patients received infusions (electrolytes, etc.), gastric mucosal protective agents and proton pump inhibitors (PPIs). The stomach tube was removed according to each patient’s condition. If patients had a fever after ESD, they were treated according to the experience of the doctors (either physical cooling, observation treatment, or use of second-generation cephalosporins for three consecutive days depending on the situation), and their temperature was tested every two hours until their temperature returned to normal. The maximum body temperature was recorded in the study. If they did not have any complications after ESD or ESE, they were permitted to return to a normal diet gradually.

### Statistical analysis

We divided the patients into two groups according to whether antibiotics were used. The variables are presented as the mean ± standard deviation (SD), the median and interquartile range (IQR) or proportion, as appropriate. Propensity score (PS) analysis was performed as a non-randomized sensitivity analysis to control and reduce the selection bias of each group. PS was estimated by using a multivariable logistic regression model with the following covariates: age, sex, diabetes, hypertension, previous abdominal surgery, lesion location, tumour size, pathology, intraoperative bleeding, operation time, en bloc resection, maximum body temperature, and stomach tube. The match ratio was 1:1, and the “nearest neighbour matching” method was used (calliper width = 0.1). The absolute standardized difference (ASD) was used to assess the balance of covariates between the two groups. An ASD < 0.1 signifies a good balance for a particular covariate. Then, we compared the fever days and hospitalization days between the two groups after matching.

The differences in baseline characteristics between the antibiotic and non-antibiotic groups were assessed using Student’s t-test for continuous variables of a normal distribution, the chi-square test or Fisher’s exact test for categorical variables, and the Wilcoxon rank-sum test for rank variables and continuous variables of an abnormally distributed, as appropriate. *p* < 0.05 was considered to be statistically significant. Statistical analyses were performed using R statistical software 3.6.1 (www.r-project.org) and IBM SPSS Statistics for Windows (V. 23.0).

## Results

### Cohort characteristics

**Fig. 1:**
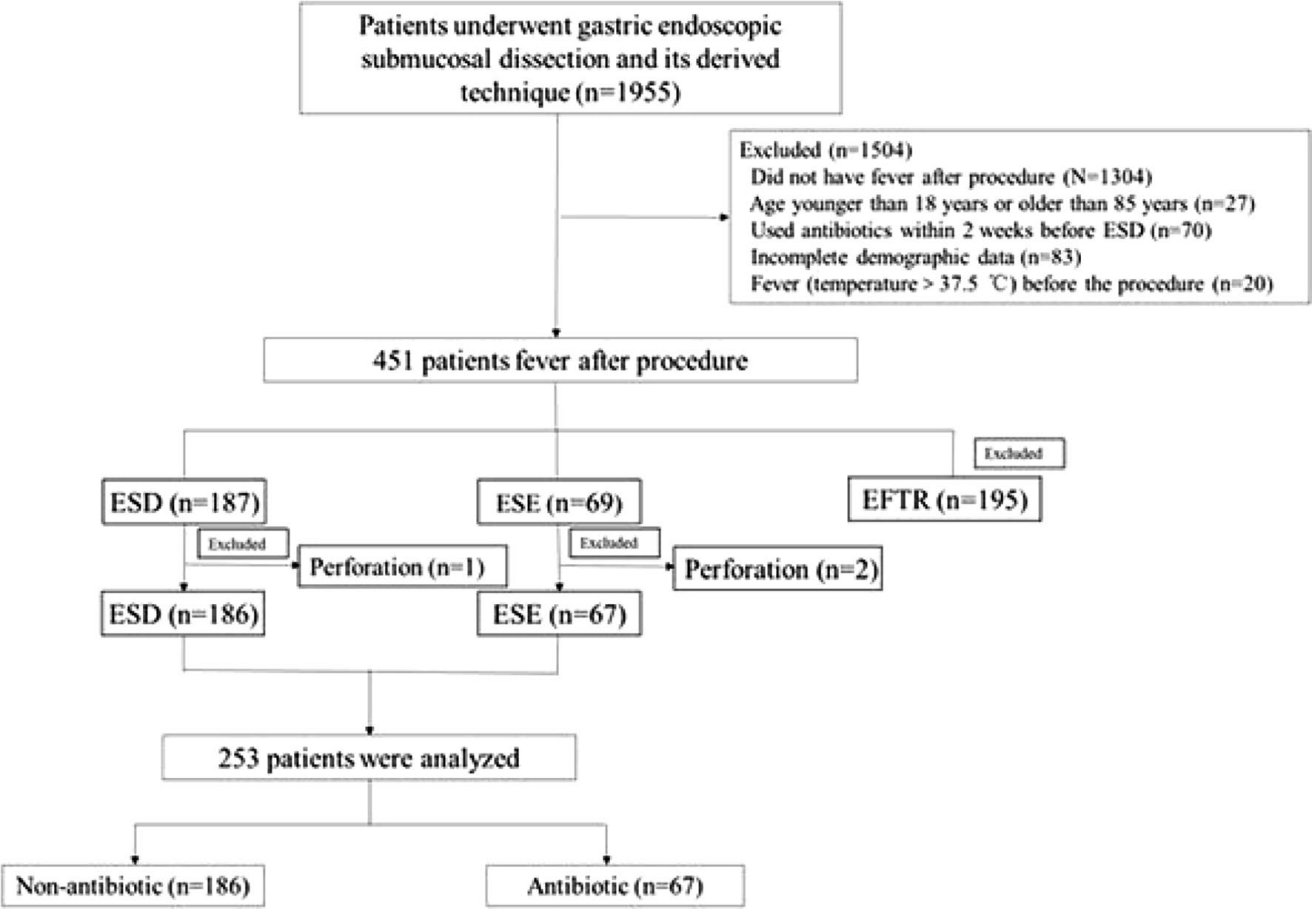
Flowchart of patients included in the present study

A total of 1955 patients who had gastric lesions underwent ESD or ESD during the study period at our centre. Of these, 253 patients (12.94%, Fig. 1) with fever and without perforation after the procedure were included in this study. Table 1 shows the baseline characteristics of the cohort. The mean age of these patients was 53.6 ± 13.4 years old, and 168 (66.4%) patients were female. The most common pathology after gastric ESD was leiomyoma (23.7%), followed by stromal tumor (17.4%).

**Table 1:** Baseline characteristics

Characteristic	N = 253
Age, mean ± SD	53.6 ± 13.4
Gender [No. (%)]	
Female	168 (66.4)
Male	85 (33.6)
Diabetes [No. (%)]	12 (4.7)
Hypertension [No. (%)]	46 (18.2)
Previous abdominal surgery [No. (%)]	34 (13.4)
Lesion location [No. (%)]	
Upper 1/3 stomach	70 (27.7)
Middle 1/3 stomach	85 (33.6)
Lower 1/3 stomach	98 (38.7)
Median tumor size (mm, IQR)	12 (8–19)
Pathology [No. (%)]	
Adenoma	34 (13.4)
Adenocarcinoma	20 (7.9)
Hyperplastic polyp	24 (9.5)
Heterotopic pancreas	26 (10.3)
Lipoma	3 (1.2)
Leiomyoma	60 (23.7)
Neuroendocrine tumor	5 (2)
Fibroma	7 (2.8)
Stromal tumor	44 (17.4)
inflammation	16 (6.3)
Other	14 (5.5)
Intraoperative bleeding [No. (%)]	81 (32)
Median operation time(min, IQR)	35 (24–47)
En bloc resection [No. (%)]	233 (92.1)
Median maximum body temperature (°C, IQR)	37.7 (37.6–38.1)
< 38.5 °C	220 (87)
≥ 38.5 °C	33 (13)
Stomach tube placement [No. (%)]	170 (67.2)

**Fig. 2:**
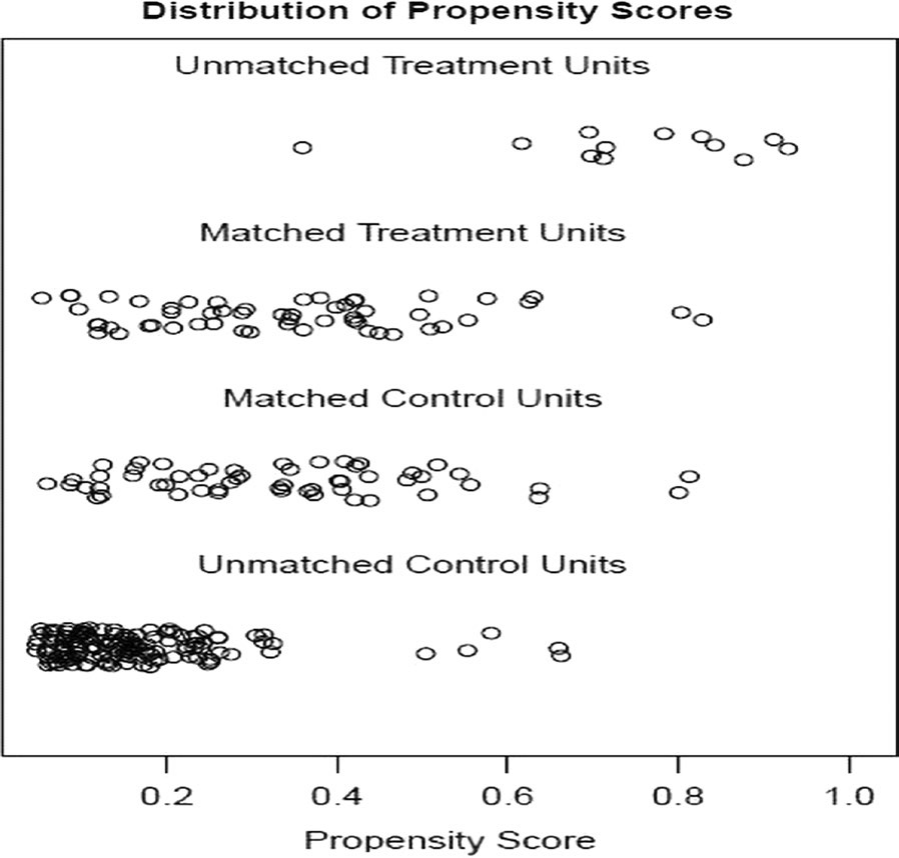
Scatter diagram of tendency distribution before and after matching

**Fig. 3:**
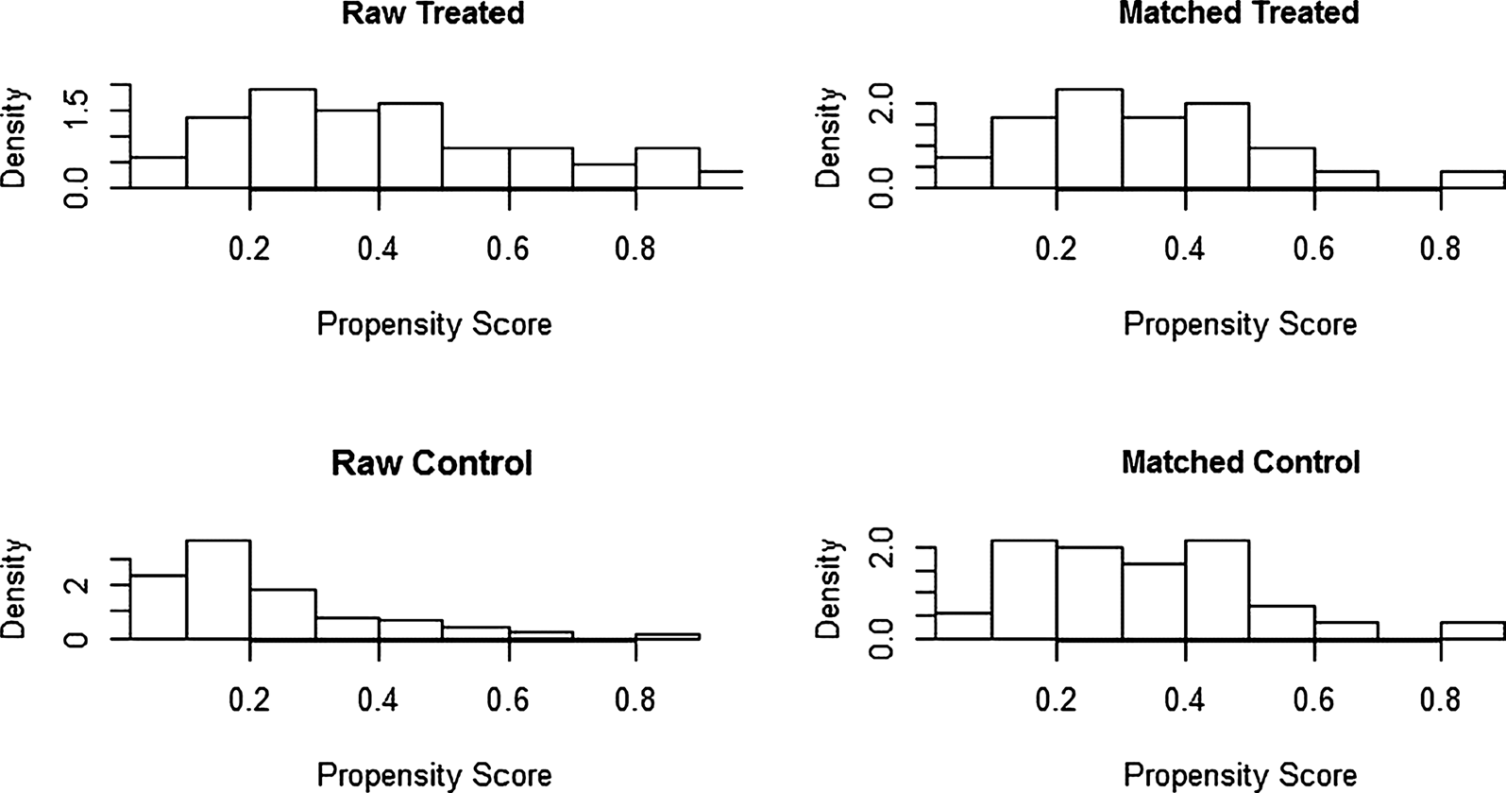
Histogram of tendency distribution before and after matching

Table 2 shows the clinical characteristics of patients before and after PS. A total of 67 patients received antibiotics when they had pyrexia after ESD or ESE, while 186 did not receive antibiotics. Before PS matching, there were significant differences in 5 factors between the 2 groups, as follows: age (*p* = 0.014), hypertension (*p* = 0.042), lesion location (*p* < 0.001), maximum body temperature (*p* < 0.001) and stomach tube (*p* = 0.008). After PS matching, a total of 110 patients were paired for the analysis. There was no significant difference in the baseline characteristics between the pairs, and the scatter diagram (Fig. 2) and histogram (Fig. 3) of the tendency distribution show good matching. The ASD for all matched covariates was < 0.1.

**Table.2:** Clinical characteristics of patients in the study before and after propensity score

	Before matching			*p*	After matching			*p*
	Non-antibiotics(n = 186)	Antibiotics (n = 67)	ASD^a^		Non-antibiotics(n = 55)	Antibiotics (n = 55)	ASD^a^	
Age, mean ± SD	52.8 ± 13.0	55.6 ± 14.5	0.686	0.014	55.9 ± 13.4	54.5 ± 14.1	0.021	0.609
Gender [No. %]			0.024	0.763			0.022	0.327
Female	125 (67.2)	143 (64.2)			31 (56.4)	37 (67.3)		
Male	61 (32.8)	24 (35.8)			24 (43.6)	18 (32.7)		
Diabetes [No. %]	8 (4.3)	4 (6)	0.014	0.738	6 (10.9)	4 (7.3)	0.011	0.742
Hypertension [No. %]	28 (15.1)	18 (26.9)	0.022	0.042	16 (29.1)	14 (25.5)	0.02	0.831
Previous abdominal surgery [No. %]	24 (12.9)	10 (14.9)	0.017	0.835	7 (12.7)	8 (14.5)	0.015	1
Lesion location [No. %]			0.24	< 0.001			0.04	0.839
Upper 1/3 stomach	48 (25.8)	22 (32.8)			18 (32.7)	16 (29.1)		
Middle 1/3 stomach	63 (33.9)	22 (32.8)			20 (36.4)	19 (34.5)		
Lower 1/3 stomach	75 (40.3)	23 (34.3)			17 (30.9)	20 (36.4)		
Median tumor size (mm, IQR)	10 (8–15)	13 (8–26)	0.596	0.127	14 (8–20)	13 (8–19)	0.044	0.507
Pathology [No. %]			0.021	0.546			0.018	0.823
Mucosa / Submucosa	142 (76.3)	51 (76.1)			41 (74.5)	43 (78.2)		
Muscular layer	44 (23.7)	16 (23.9)			14 (25.5)	12 (21.8)		
Intraoperative bleeding [No. %]	54 (29)	27 (40.3)	0.024	0.095	22 (40)	21 (38.2)	0.021	1
Median operation time (min, IQR)	33 (23–43)	37 (24.5–56.5)	0.763	0.076	37 (30–51)	37 (21.5–50.5)	0.078	0.183
En bloc resection [No. %]	173 (93)	60 (89.6)	0.014	0.428	54(98.2)	50 (90.9)	0.011	0.206
Median maximum body temperature (°C, IQR)	37.6 (37.6–38)	38 (37.8–38.6)	0.023	< 0.001	37.9 (37.6–38.4)	38 (37.7–38.4)	0.02	0.523
< 38.5 °C	174 (93.5)	46 (68.7)			45 (81.8)	43 (78.2)		
≥ 38.5 °C	12 (6.5)	21 (31.3)			10 (18.2)	12 (21.8)		
Stomach tube placement [No. %]	124 (66.7)	46 (68.7)	0.024	0.008	39 (70.9)	38 (69.1)	0.023	1

^a^The absolute standardized difference (ASD) was used to assess the balance of covariates between the two groups. Variables with an ASD > 0.10 is considered to be imbalance

### Comparison between the non-antibiotic group and the antibiotic group

After PS matching, the selection bias of each group was reduced and controlled. The fever durations between the two groups were compared by rank sum test, and the hospitalization time between the two groups was compared by Mann–Whitney rank sum test. The rank average of fever duration in the antibiotic group was higher than that in the non-antibiotic group, but the results were not significant (60.05 vs 48.95, *p* = 0.12). However, the median length of hospital stay in the antibiotic group was longer than that in the non-antibiotic group (8 vs 7, *p* = 0.007) (Table 3).

**Table 3:** Comparison between the two groups (adjustment through PS regression)

	Non-antibiotic	Antibiotic	*p*
Fever time^a^	48.95^b^	62.05^b^	0.12
Hospitalization stay [days, median(IQR)]	7 (6–9)	8 (7–9)	0.007

^a^Fever time was recorded as rank variables and comparison was made by Wilcoxon rank sum test

^b^Average rank

In the subgroup analysis, when maximum body temperature was < 38.5 °C, the median hospitalization duration of the antibiotic group was still longer than that of the non-antibiotic group (8 vs 7, *p* = 0.038), and the comparison of fever duration was still nonsignificant (39.86 vs 49.33, *p* = 0.33) (Table 4). In the subgroup with a temperature ≥ 38.5 °C, the comparison between the two groups in the median hospitalization duration in days (6.5 vs 8, *p* = 0.14) and the rank mean of fever duration in days (9.4 vs 13.25, *p* = 0.156) were not significant.

**Table 4:** Subgroup analysis of the two groups (adjustment through PS regression)

		Non-antibiotic	Antibiotic	*p*
< 38.5 °C	Fever time^a^	39.86^b^	49.33^b^	0.33
	Hospitalization stay [days, median(IQR)]	7 (6–9)	8 (7–10)	0.038
≥ 38.5 °C	Fever time^a^	9.4^b^	13.25^b^	0.156
	Hospitalization stay [days, median(IQR)]	6.5 (6–9)	8 (7–12)	0.14

^a^Fever time was recorded as rank variables and comparison was made by Wilcoxon rank sum test

^b^Average rank; hospitalization was abnormal distribution

## Discussion

Fever is one of the common complications after ESD and ESE, but the mechanism of fever development after ESD and ESE is still unclear. Previous studies have indicated that fever after ESD and ESE may be related to wound exposure and bacteraemia [10]. Some guidelines have recommended prophylactic use of antibiotics after ESD or ESE [14, 15]. However, Kato et al. found that the incidence of bacteraemia after ESD was low [11]. Lee et al. found that bacteraemia after gastric ESD was temporary [9]. Other studies also concluded that prophylactic antibiotics might be unnecessary for patients with gastric ESD or ESE [8, 10]. Since fever after ESD or ESE may be non-infectious, the necessity of antibiotics for fever after ESD or ESE is confusing, especially for those patients without perforation after procedure. The aim of this study was to analyse the need for antibiotics for postoperative fever in patients without perforation.

Before procedure, patients need complete preoperative examination, including CT, EUS, laboratory examination and so on. Hence, in our study, the median hospitalization stay is 7 (6–9) in non-antibiotic group and 8 (7–10) antibiotic group. In the present study, the use of antibiotics did not shorten the duration of fever but increased the duration of hospitalization. This was also the case in the subgroup analysis of individuals with a temperature < 38.5 °C. There was no difference in the duration of fever between the patients with a temperature above 38.5 °C who used antibiotics and those who did not use antibiotics. In addition, there was no significant difference in the number of days spent in the hospital between the two groups, which might be related to the small sample size. The mechanism of fever development after ESD and ESE is not clear. In clinical situation, some doctors will empirically use antibiotics for patients with fever after an ESD or ESE procedure. However, in this study, the use of antibiotics did not reduce the duration of fever and even increased the hospitalization stay. In addition, the use of antibiotics will also increase the cost of hospitalization and may cause adverse reactions to antibiotics, such as allergies, drug resistance and secondary infection [16–18]. We hypothesized that pyrexia after ESD or ESE is a physiological febrile response similar to that occurring after surgery [19]. The fever may be due to the release of inflammatory cytokines from macrophages, endothelial cells and the reticuloendothelial system after tissue damage, and these cytokines cause the elevation of the thermoregulatory set point for body temperature [20, 21].

Although this was a retrospective study, our research had a large sample size, and we compared the fever time between patients in the antibiotic and non-antibiotic groups. In addition, we performed PS matching to minimize bias. Importantly, few studies have evaluated the necessity of using antibiotics for fever in patients without perforation after ESD or ESE.

There were some limitations of the present study. Firstly, the present study was a single-centre retrospective study. The findings of the present study need to be validated by multicentre prospective studies. Secondly, the use of antibiotics was according to the experience of the doctors, which may potentially introduce a source of bias: such as the most severe cases received antibiotics and the mild ones did not. However, this study used PS analysis to control and reduce such bias. Hence, this problem can be neglected. Thirdly, this study excluded patients with serious comorbidities, therefore, this study's results cannot be uniformly applied to all the patients and further research is needed in the future.

## Conclusion

In conclusion, doctors can choose observation treatment and antibiotics may be unnecessary for fever in patients without perforation and without serious comorbidities after ESD or ESE, for antibiotics may not have much effect on fever after ESD or ESE but will increase hospitalization duration.

## Data Availability

The datasets used and/or analysed during the current study are available from the corresponding author on reasonable request.

## Abbreviations

ESD: Endoscopic submucosal dissection
PS: Propensity score
EUS: Endoscopic ultrasound
CT: Computerized tomography
APC: Argon plasma coagulation
MP: Muscularis propria
ESE: Endoscopic submucosal excavation
OTSCs: Over-the-scope clip system
PPIs: Proton pump inhibitors
SD: Standard deviation
IQR: Interquartile range
ASD: Absolute standardized difference
